# Association between fluid balance, muscle strength, and muscle quality in critically ill patients: a retrospective observational study

**DOI:** 10.1186/s40635-026-00966-6

**Published:** 2026-08-28

**Authors:** Nicolas Arancibia-Ramirez, Yorschua Jalil Contreras, Alexis Silva, L. Felipe Damiani, Jorge Molina, Cheryl E. Hickmann

**Affiliations:** 1 Servicio de Medicina Física y Rehabilitación, Clínica Alemana de Santiago, Santiago, Chile; 2 Departamento de Kinesiología, Escuela de Ciencias de la Salud, Facultad de Medicina, Pontificia Universidad Católica de Chile, Santiago, Chile; 3 Adult Critical Care Unit, Hospital Regional de Ñuble, Chillán, Chile; 4 Servicio de medicina física y rehabilitación Hospital Regional de Ñuble, Chillán, Chile; 5 Programa de especialidad médica en anestesiología y reanimación, Universidad Católica de la Santísima Concepción, Chillán, Chile; 6 Carrera de Kinesiología, Facultad de Medicina, Clínica Alemana, Universidad del Desarrollo, Santiago, Chile; 7 Department of Movement Sciences and Exercise & Movement Laboratory, HEPH- Condorcet, Tournai, Belgium; 8 Intensive Care Unit, CHU-HELORA University Hospital, Mons, Belgium; 9 Condorcet EpiCURA Research Unit (URICE), EpiCURA Hospital Center, Hornu, Belgium

**Keywords:** Fluid balance, Intensive Care Unit–acquired weakness, Muscle ultrasound, Heckmatt scale, Muscle strength, Critically ill patients

## Abstract

**Background:**

Intensive Care Unit–acquired weakness (ICUAW) is a frequent complication that significantly impairs functional recovery in critically ill patients. Fluid overload during critical illness has been associated with adverse outcomes, but its relationship with peripheral muscle quality and strength remains uncertain. This study aimed to evaluate the association between cumulative fluid balance, ultrasound-assessed muscle quality, and muscle strength in critically ill patients.

**Patients and methods:**

We conducted a retrospective analysis of routinely collected data between May and October 2024 in adult ICU patients who were deeply sedated, mechanically ventilated, and had an ICU length of stay ≥24 hours. Muscle and subcutaneous tissue measurements were obtained using ultrasound of the vastus intermedius and rectus femoris within the first 24 hours of ICU admission (day 1) and at clinical awakening. Muscle quality was assessed at both time points using the Heckmatt scale. Global muscle strength was assessed only at clinical awakening using the Medical Research Council Sum Score (MRC-SS), once patients were able to follow commands. Patients without complete ultrasound measurements at both time points were excluded from the final analysis.

**Results:**

Eighty patients were included (mean age 51 ± 14 years; APACHE II score 18 ± 5). The main admission diagnoses were predominantly respiratory and septic conditions. Mean cumulative fluid balance at awakening was 2400 ± 800 mL. From ICU admission to awakening, quadriceps thickness decreased, whereas subcutaneous tissue thickness increased and muscle quality deteriorated, as reflected by higher Heckmatt scores. At awakening, cumulative fluid balance showed a strong inverse correlation with global muscle strength (rho = −0.70, p < 0.001) and a moderate positive correlation with subcutaneous tissue thickness. Muscle quality assessed by the Heckmatt scale was moderately inversely correlated with MRC-SS (rho = −0.43, p< 0.001). In multivariable analysis adjusted for age, disease severity, and duration of mechanical ventilation, cumulative fluid balance remained independently associated with lower muscle strength at awakening.

**Conclusions:**

Qualitative muscle ultrasound assessment using the Heckmatt scale provides complementary information to muscle thickness measurements. The observed association between cumulative fluid balance and poorer muscle quality suggests that fluid status should be considered when interpreting qualitative muscle ultrasound findings in critically ill patients.

## Introduction

Intensive Care Unit–acquired weakness (ICU-AW) is a frequent complication in critically ill patients, characterized by loss of muscle mass and quality, with a direct impact on functional recovery, prolonged mechanical ventilation, increased hospital length of stay, and mortality [1, 2]. Its development is multifactorial and involves systemic inflammation, prolonged immobilization, exposure to corticosteroids and neuromuscular blocking agents, and metabolic stress during critical illness [3, 4].

Fluid administration is an integral component of the management of many critically ill patients, particularly during the early phase of hemodynamic stabilization. Although the amount of fluid administered varies according to the underlying disease and clinical condition, positive cumulative fluid balance is frequently observed in mechanically ventilated ICU patients because of fluid resuscitation when clinically indicated, maintenance therapy, medications, nutritional support, and reduced fluid elimination. Persistent positive fluid balance has been associated with adverse outcomes, including organ dysfunction, prolonged mechanical ventilation, delayed recovery, and mortality [5–9]. Beyond these systemic consequences, fluid accumulation may also contribute to skeletal muscle dysfunction through mechanisms such as interstitial edema, impaired microcirculatory perfusion, increased intramuscular pressure, and extracellular matrix remodeling [10, 11]. These alterations may aggravate muscle dysfunction and contribute to ICU-acquired weakness and post-intensive care syndrome (PICS) [12, 13].

Early identification of muscle alterations in critically ill patients remains challenging. Conventional assessments, including physical examination and functional testing, are often not feasible during the acute phase because of deep sedation and mechanical ventilation. In this context, musculoskeletal ultrasound has emerged as a non-invasive, bedside, and reproducible tool for evaluating skeletal muscle structure and quality in critically ill patients [14, 15]. The Heckmatt scale, originally developed for neuromuscular disorders, classifies muscle quality according to echogenicity and fascial distinction and has recently been explored in critically ill populations [16–18].

Emerging evidence suggests that muscle quality may be more closely associated with functional recovery than muscle size alone [19, 20].

However, the relationship between cumulative fluid balance, ultrasound-assessed muscle quality, and muscle strength at awakening remains poorly understood.

Therefore, this retrospective observational study aimed to evaluate the association between cumulative fluid balance, muscle quality assessed by ultrasound, and global muscle strength measured at awakening using the Medical Research Council Sum Score (MRC-SS) in critically ill patients.

## Methods

### Study design and ethical considerations

A retrospective observational study was conducted at the Intensive Care Unit of Clínica Alemana de Santiago, Chile. The protocol was approved by the Institutional Ethics Committee (IRB #2012-53). Given the retrospective nature of the study, the institutional ethics committee approved a waiver of informed consent. All procedures were conducted in accordance with applicable ethical standards and regulations for research involving human data, including data protection and confidentiality, and in compliance with the Declaration of Helsinki and its later amendments.

### Study population

Patients admitted between May and October 2024 with available routine muscle ultrasound images at both ICU admission and clinical awakening were eligible. Exclusion criteria included pre-existing neuromuscular disorders, limb amputations, documented limitations of therapeutic effort upon admission, or poor-quality ultrasound images precluding analysis.

Adult patients (≥ 18 years) undergoing invasive mechanical ventilation for at least 24 h were included. At ICU admission, and as part of routine clinical management during the early phase of critical illness, all included patients were deeply sedated, with a Richmond Agitation–Sedation Scale (RASS) score of − 4 to − 5.

After the acute phase, sedation was progressively reduced according to clinical indications. Clinical awakening was defined as the first assessment at which patients were able to follow commands, based on at least four correct responses on the 5 Simple Questions (S5Q). The S5Q consists of five standardized commands (open/close your eyes, look at me, open your mouth and protrude your tongue, nod your head, and raise your eyebrows) used to determine whether patients are able to cooperate with clinical assessment. At this time point, global muscle strength, routinely assessed in clinical practice using the Medical Research Council Sum Score (MRC-SS), was recorded from medical records [21].

Ultrasound examinations were performed as part of routine clinical care. All ultrasound images were digitally stored in the institutional imaging archive and retrospectively reviewed for the purposes of this study. Clinical variables, including cumulative fluid balance and Medical Research Council Sum Score (MRC-SS), were extracted from the institutional electronic medical records.

### Clinical and demographic variables

Age, sex, admission diagnosis, Acute Physiology and Chronic Health Evaluation II (APACHE II) score, duration of mechanical ventilation, and ICU length of stay were extracted from the electronic medical records. Vasopressor use during ICU stay was recorded as a dichotomous variable (yes/no) and used as a surrogate marker of circulatory shock. The duration of vasopressor therapy was also recorded (days). Delirium at clinical awakening was assessed using the Confusion Assessment Method for the ICU (CAM-ICU) by trained ICU staff according to the unit’s routine clinical practice.

### Ultrasound assessments

Muscle assessments were performed using B-mode ultrasound with a SonoSite M-Turbo ultrasound system (FUJIFILM SonoSite Inc., Bothell, WA, USA) equipped with a high-frequency linear-array transducer (6–13 MHz). Structural examination of the vastus intermedius (VI) and rectus femoris (RF) muscles was performed at the 1/2 point between the anterior superior iliac spine and the superior border of the patella, with the patient in the supine position and the lower limb relaxed. Muscle thickness was measured from the superficial fascia of the RF to the deep fascia of the VI. Subcutaneous tissue thickness was measured from the skin to the superficial fascia of the RF. To minimize measurement bias, minimal transducer compression was applied during image acquisition. Imaging parameters, including transducer frequency, gain, depth, and focal zone, were kept as consistent as possible throughout the study according to a standardized institutional acquisition protocol, with only minimal adjustments when necessary to optimize visualization of the muscle anatomy. Muscle quality was assessed qualitatively using the Heckmatt scale, as previously described [16], which grades muscle echogenicity and fascial distinction from 1 (normal) to 4 (severely abnormal). Additionally, quantitative grayscale analysis was performed offline using ImageJ software (National Institutes of Health, Bethesda, MD, USA) [22]. For each stored ultrasound image, a standardized region of interest (ROI) was manually selected within the muscle belly while avoiding fascia and bone, and mean grayscale values were quantified on an 8-bit scale ranging from 0 (black) to 255 (white). All ultrasound examinations were performed as part of routine clinical care by a physiotherapist experienced in musculoskeletal ultrasound, following a standardized institutional acquisition protocol. The stored ultrasound images were subsequently reviewed for the purposes of this retrospective study.

Two assessments were performed: (1) within the first 24 h of ICU admission (day 1) and (2) at awakening (5 Simple Questions;5SQ ≥ 4).

### Muscle strength assessment

Global muscle strength was assessed at clinical awakening using the Medical Research Council Sum Score (MRC-SS), which evaluates six bilateral muscle groups with a total score ranging from 0 to 60. Muscle strength assessments were performed by one of several physiotherapists trained in MRC-SS evaluation according to the unit’s standardized clinical practice [21].

### Fluid balance

Cumulative fluid balance (mL) was calculated as the sum of the daily differences between total fluid intake and output (including urine output, drains, and ultrafiltration) from ICU admission until clinical awakening.

Fluid balance data were retrospectively extracted from the institutional electronic medical record. Daily fluid intake and output were prospectively documented by ICU nursing staff on standardized nursing fluid balance charts as part of routine clinical care. Daily and cumulative fluid balance were routinely calculated from these nursing records according to institutional practice and subsequently retrieved for this retrospective study.

### Statistical analysis

Continuous variables were described as mean ± standard deviation (SD) or median and interquartile range (IQR) according to their distribution (assessed with the Shapiro–Wilk test). Categorical variables were expressed as frequencies and percentages.

As normality assumptions were not met for paired measurements, the Wilcoxon signed-rank test was used to compare measurements between day 1 and awakening. Correlations between variables were assessed using Spearman’s correlation coefficient. A permutation test was performed to confirm the statistical significance of the correlation between cumulative fluid balance and muscle quality (Heckmatt scale). Additionally, a multivariable linear regression model was performed to identify factors independently associated with muscle strength (MRC-SS) at awakening. MRC-SS was specified as the dependent variable, whereas cumulative fluid balance, age, APACHE II score, and duration of mechanical ventilation were included as independent variables. Variables were entered simultaneously using the enter method. Age, APACHE II score, duration of mechanical ventilation, and cumulative fluid balance were selected a priori based on their established clinical relevance as potential determinants of ICU-acquired weakness and muscle function. These variables were chosen because advanced age, greater illness severity, prolonged mechanical ventilation, and positive fluid balance have all been associated with impaired muscle function and poorer outcomes in critically ill patients. Multicollinearity was assessed using variance inflation factors (VIF), with all VIF values below 2, indicating no evidence of problematic multicollinearity. Model assumptions were assessed by visual inspection of residuals. A p-value < 0.05 was considered statistically significant.

## Results

**Fig. 1:**
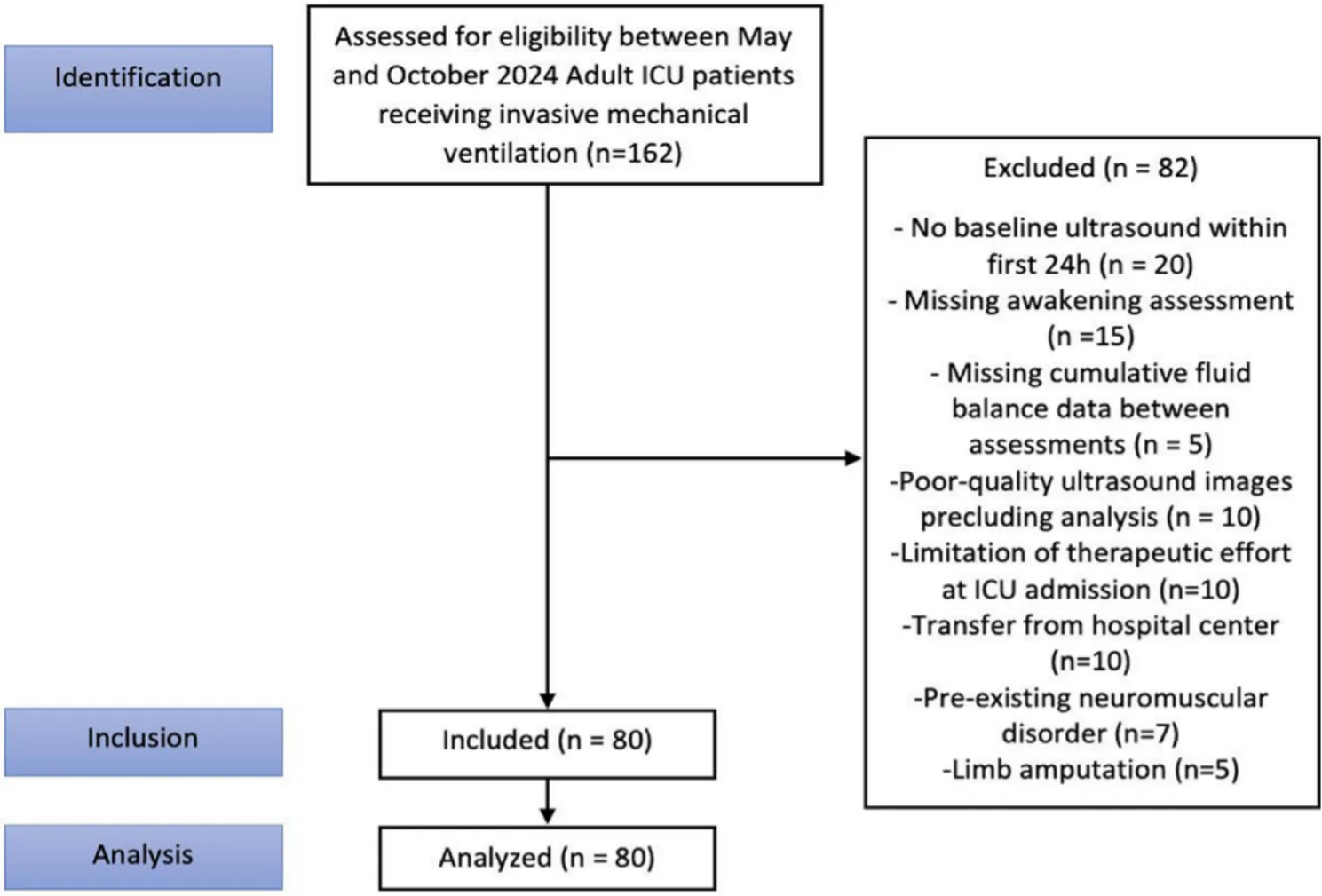
Flow diagram of patient selection and inclusion

During the study period, a total of 80 critically ill patients meeting the inclusion criteria were included in the analysis (Fig. 1). Baseline demographic and clinical characteristics are summarized in Table 1 (mean age 51 ± 14 years; APACHE II score 18 ± 5). Respiratory and septic conditions accounted for 27.5% and 18.7% of admission diagnoses, respectively.

**Fig. 2:**
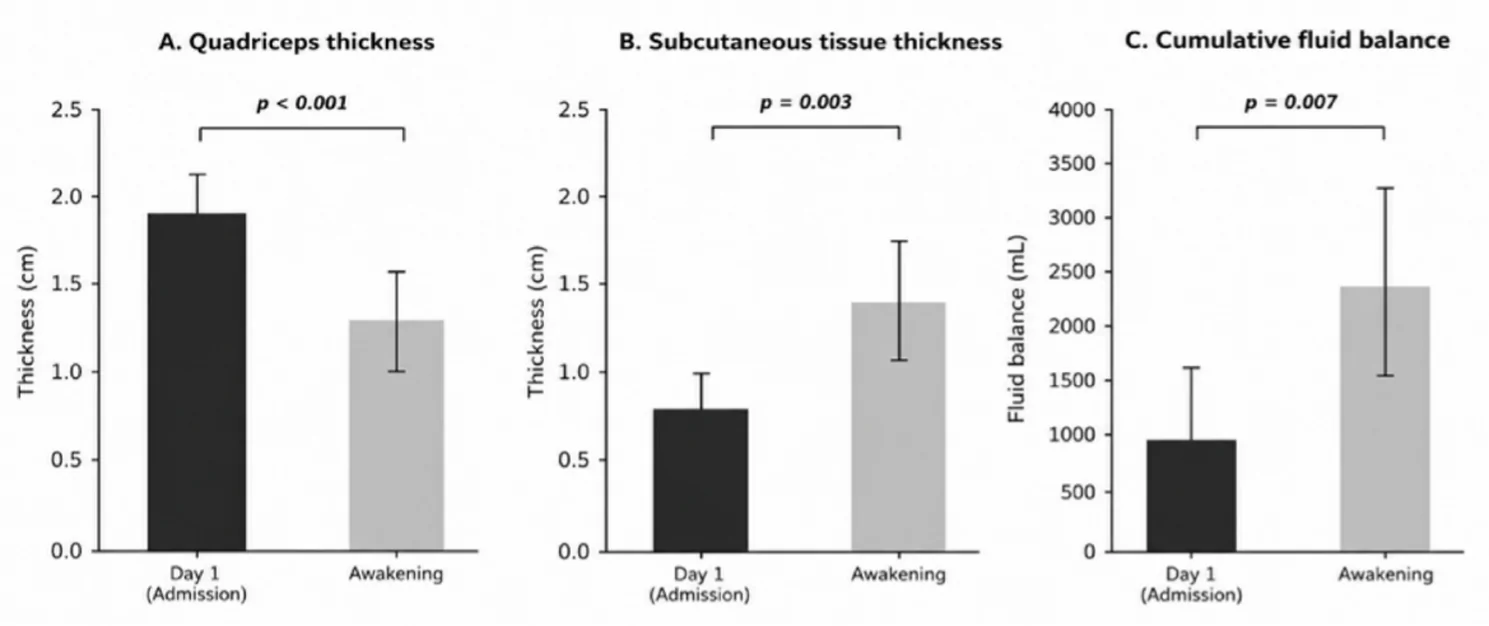
Changes in muscle tissue and fluid balance measurements between ICU admission (Day 1) and awakening. Panel **A**: Quadriceps thickness (cm). Panel **B**: Subcutaneous tissue thickness (cm). Panel **C**: Cumulative fluid balance (mL)

From ICU admission to awakening (5.0 ± 3.0 days), quadriceps thickness decreased from 1.9 ± 0.2 cm to 1.3 ± 0.3 cm, whereas subcutaneous tissue thickness increased from 0.8 ± 0.2 cm to 1.4 ± 0.3 cm. Cumulative fluid balance increased from 950 ± 600 mL at ICU admission to 2400 ± 800 mL at awakening. These changes are summarized in Fig. 2.

At awakening, mean muscle strength assessed using the Medical Research Council Sum Score (MRC-SS) was 40 ± 10 points. ICU-acquired weakness (MRC-SS < 48) was present in 63 of 80 patients (78.8%), whereas severe weakness (MRC-SS < 36) was observed in 27 patients (33.8%).

Table 2 summarizes the univariate and multivariate linear regression analyses evaluating factors associated with muscle strength at awakening. In multivariable analysis, cumulative fluid balance remained independently associated with lower MRC-SS (β = −0.35, 95% CI − 0.62 to − 0.08; *p* = 0.01).

**Fig. 3:**
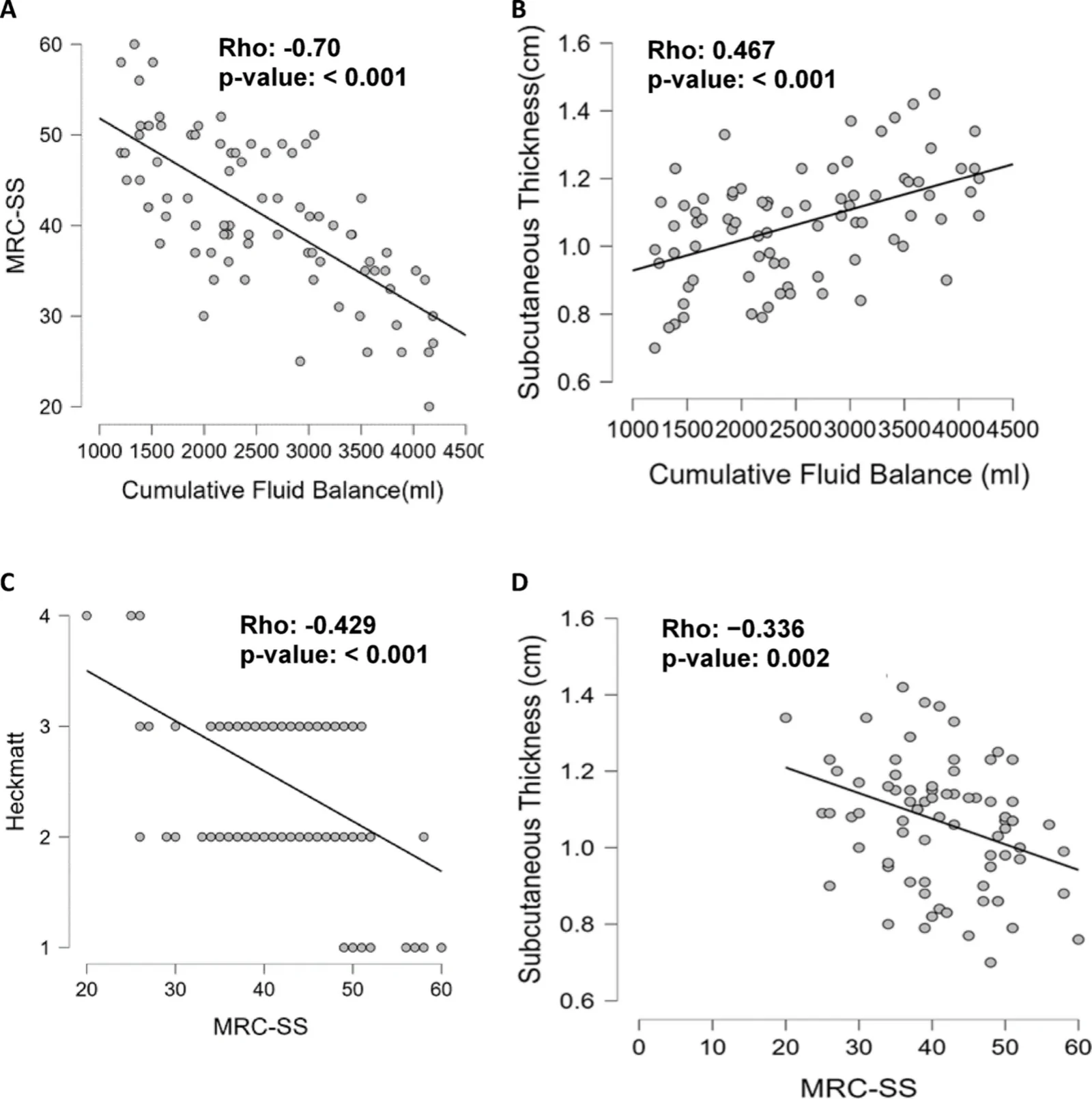
Associations between cumulative fluid balance, muscle quality, subcutaneous tissue thickness, and global muscle strength at awakening. 3**A**: Scatter plot illustrating the inverse association between cumulative fluid balance (mL) accumulated from ICU admission to awakening and global muscle strength assessed by the Medical Research Council Sum Score (MRC-SS) at awakening; 3**B**: Scatter plot showing the relationship between cumulative fluid balance and subcutaneous tissue thickness measured by ultrasound at clinical awakening; 3**C**: Scatter plot illustrating the inverse association between muscle quality assessed by the Heckmatt score and global muscle strength evaluated by the Medical Research Council Sum Score (MRC-SS) at awakenin; 3**D**: Scatter plot showing the relationship between global muscle strength assessed by the Medical Research Council Sum Score (MRC-SS) and subcutaneous tissue thickness measured by ultrasound at clinical awakening

A strong inverse correlation was observed between cumulative fluid balance and global muscle strength at awakening (rho = − 0.70, *p* < 0.001; Fig. 3A). Cumulative fluid balance was moderately positively correlated with subcutaneous tissue thickness (rho = 0.47, *p* < 0.001; Fig. 3B).

Muscle quality assessed by the Heckmatt score showed a moderate inverse correlation with MRC-SS (rho = − 0.43, *p* < 0.001; Fig. 3C). Subcutaneous tissue thickness was also inversely correlated with muscle strength (rho = − 0.34, *p* = 0.002; Fig. 3D).

**Fig. 4:**
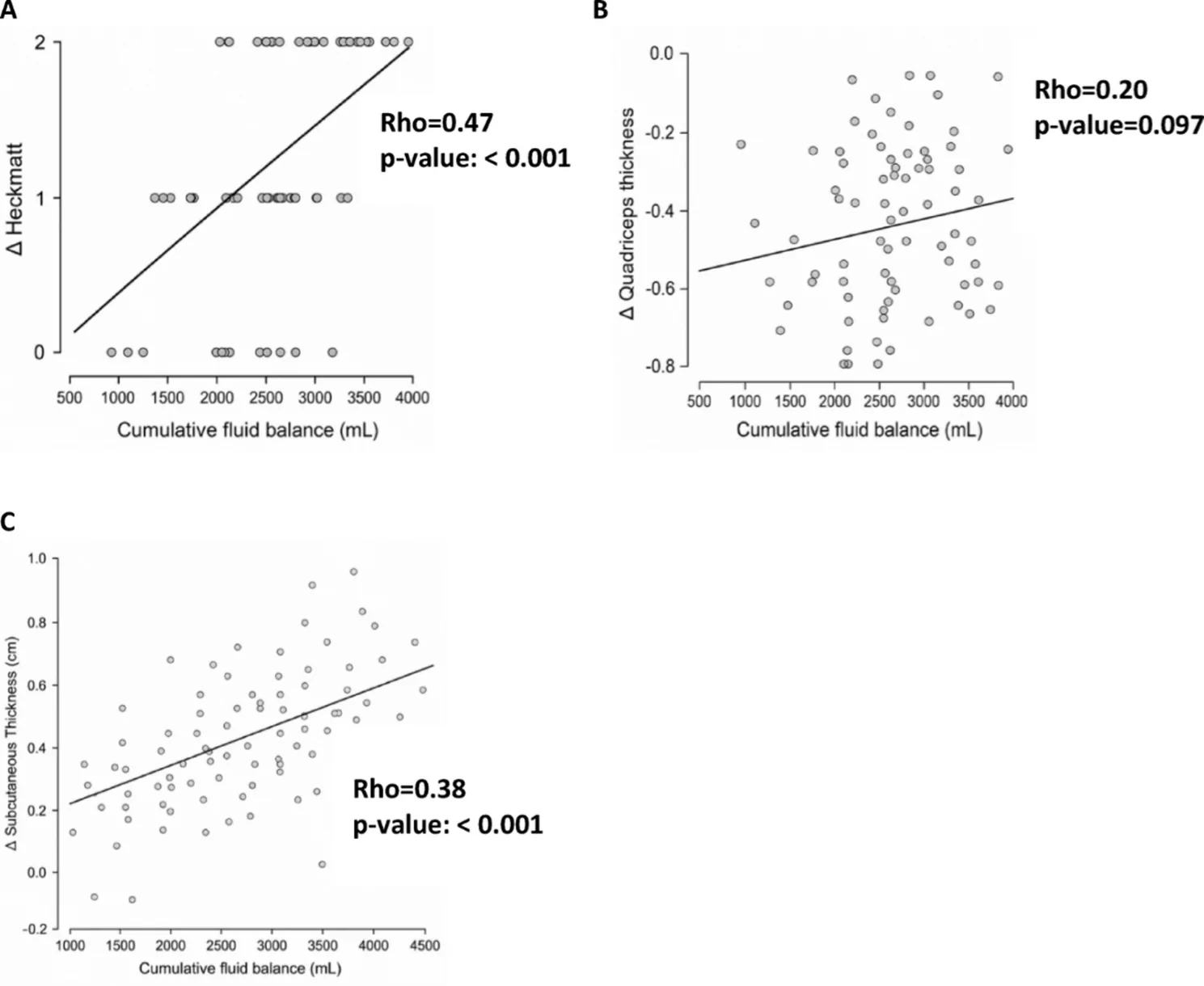
Exploratory associations between cumulative fluid balance and longitudinal changes in ultrasound-derived parameters from ICU admission to awakening. 4**A**: Δ Heckmatt score. 4**B**: Δ Quadriceps muscle thickness. 4**C**: Δ Subcutaneous tissue thickness. Scatter plots show Spearman’s correlation coefficients (ρ) and corresponding p-values for each analysis

As an exploratory analysis, cumulative fluid balance was correlated with the longitudinal changes in ultrasound-derived parameters from ICU admission to awakening. Cumulative fluid balance demonstrated a moderate positive correlation with the change in Heckmatt score (Rho = 0.47, *p* < 0.001) and with the change in subcutaneous tissue thickness (Rho = 0.38, *p* < 0.001). In contrast, only a weak, non-significant correlation was observed with the change in quadriceps muscle thickness (Rho = 0.20, *p* = 0.097) (Fig. 4).

**Fig. 5:**
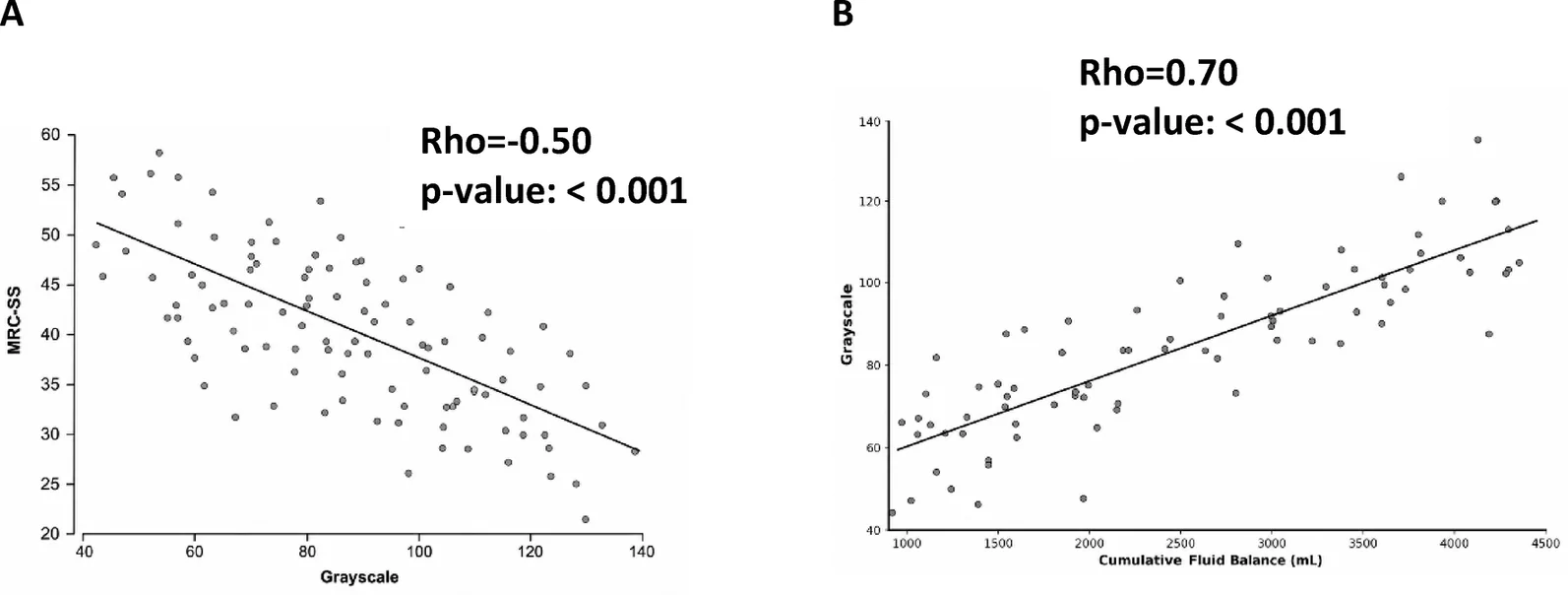
Associations between quantitative grayscale values and clinical outcomes. 5**A**: Correlation between grayscale values measured using ImageJ and muscle strength (MRC-SS). 5**B**: Correlation between grayscale values and cumulative fluid balance. Scatter plots show Spearman’s correlation analyses

Additionally, quantitative grayscale analysis was performed using ImageJ software. Grayscale values were moderately inversely correlated with MRC-SS (Rho = − 0.50, *p* < 0.001) and strongly positively correlated with cumulative fluid balance (Rho = 0.70, *p* < 0.001) (Fig. 5).

A sensitivity analysis using the modified 3-point Heckmatt classification, in which grades 2 and 3 were combined according to the simplified classification previously proposed by Puthucheary et al. and subsequently supported by recent psychometric validation demonstrating improved scoring accuracy and measurement properties of the scale [23], yielded comparable results to those obtained with the original 4-point scale, supporting the robustness of the observed association between the Heckmatt score and MRC-SS (Supplementary Table S1).

**Table 1:** Baseline characteristics of the study population

Variable	Value
Age, years^*^	51 ± 14
Male sex^τ^	46 (57.5%)
APACHE II score^*^	18 ± 5
Days from ICU admission to awakening^*^	5.0 ± 3.0
ICU length of stay, days^*^	8.0 ± 4.0
Invasive mechanical ventilation, days^*^	5.0 ± 2.0
**Ventilation mode at admission** ^**τ**^	
– Pressure-controlled ventilation (PCV)	36 (45.0%)
– Volume-controlled ventilation (VCV)	20 (25.0%)
– Assisted modes (PSV)	24 (30.0%)
**Sedation level at ICU admission (RASS)** ^**τ**^	
– Deep sedation (RASS ≤ − 4)	62 (77.5%)
– Moderate sedation (RASS − 3 to − 2)	14 (17.5%)
– Light sedation/calm (RASS − 1 to 0)	4 (5.0%)
**Sedative agents used during ICU stay** ^**τ**^	
– Propofol	54 (67.5%)
– Benzodiazepines	18 (22.5%)
– Dexmedetomidine	16 (20.0%)
**Other ICU-related clinical characteristics**	
Neuromuscular blocking agents (any)^τ^	24 (30.0%)
Vasopressor use (any)^τ^	42 (52.5%)
Duration of vasopressor support (days)^#^	2 [1–3]
Any in-bed mobilization during ICU stay^τ^	50 (62.5%)
Early out-of-bed mobilization before clinical awakening (sitting, standing, or transfer to chair)	12 (15.0%)
Delirium at awakening (CAM-ICU positive)^τ^	24 (30.0%)
**Admission diagnosis** ^**τ**^	
– Pneumonia	22 (27.5%)
– Sepsis	15 (18.8%)
– COPD exacerbation	12 (15.0%)
– ARDS	10 (12.5%)
– Heart failure	8 (10.0%)
– Other diagnoses	9 (11.3%)
– Major postoperative surgery	4 (5.0%)

Data are presented as *mean ± standard deviation (SD), #median [interquartile range], or τnumber (%), as appropriate

APACHE II = Acute Physiology and Chronic Health Evaluation II; PCV = pressure-controlled ventilation; VCV = volume-controlled ventilation; PSV = pressure support ventilation; RASS = Richmond Agitation–Sedation Scale; CAM-ICU = Confusion Assessment Method for the ICU

Vasopressor use was recorded as a surrogate marker of circulatory shock

**Table 2:** Linear regression analysis of factors associated with muscle strength (MRC-SS) at awakening (*n* = 80)

Variable	Univariate β (95% CI)	*p*-value	Multivariate β (95% CI)	*p*-value
Cumulative fluid balance (per 1 L)	–0.44 (–0.62 to − 0.26)	< 0.001	–0.35 (–0.62 to − 0.08)	0.01
Muscle quality (Heckmatt score)	–2.10 (–3.90 to − 0.30)	0.02	–1.20 (–3.10 to 0.70)	0.21
Age (years)	–0.12 (–0.22 to − 0.02)	0.02	–0.05 (–0.16 to 0.06)	0.37
APACHE II score	–0.28 (–0.52 to − 0.04)	0.02	–0.11 (–0.36 to 0.14)	0.39
Days on mechanical ventilation	–0.85 (–1.55 to − 0.15)	0.02	–0.42 (–1.20 to 0.36)	0.29

β coefficients are presented with 95% confidence intervals (CI)

Univariate linear regression analyses were performed for each variable independently

Multivariate linear regression included age, APACHE II score, days on mechanical ventilation, and cumulative fluid balance based on clinical relevance and univariate significance

Cumulative fluid balance was expressed per 1-liter increase

Muscle strength was assessed using the Medical Research Council Sum Score (MRC-SS) at awakening

A p-value < 0.05 was considered statistically significant

## Discussion

The present study evaluated the association between cumulative fluid balance, changes in peripheral muscle quality assessed by ultrasound, and global muscle strength at awakening in mechanically ventilated critically ill patients. Our findings suggest that higher cumulative fluid balance was associated with poorer muscle quality and lower global muscle strength at the time of functional assessment. However, given the observational design, these findings should be interpreted as associations rather than evidence of a causal relationship.

In our cohort, clinical awakening occurred at a mean of 5 ± 2 days after ICU admission. During this period, quadriceps thickness decreased from 1.9 to 1.3 cm (approximately 32%), accompanied by a shift toward higher Heckmatt scores, indicating worsening muscle quality. These changes occurred in parallel with increased cumulative fluid balance.

Importantly, muscle deterioration in critically ill patients is a multifactorial process. Factors such as prolonged mechanical ventilation, systemic inflammation, nutritional deficits, and immobility contribute to muscle wasting and dysfunction [24]. Therefore, fluid accumulation should be interpreted as a potential contributing factor or marker of illness severity rather than an isolated causal mechanism.

Nevertheless, the observed association between cumulative fluid balance, muscle quality, and muscle strength is biologically plausible and may reflect several mechanisms previously described in critical illness, including interstitial edema, impaired microcirculation, altered tissue composition, and the overall severity of illness. However, the present study was not designed to determine the relative contribution of these mechanisms or to establish causality [25].

Early muscle loss in critical illness has been well described. In the prospective study by Puthucheary et al. [26], conducted in a mixed ICU population with a mean APACHE II score of 23.5, rectus femoris cross-sectional area decreased by 12–15% at day 7 and up to 17–18% by day 10 of mechanical ventilation. In comparison, the magnitude of percentage reduction observed in our cohort at approximately day 5 was even greater.

However, unlike that study, which focused primarily on quantitative morphologic changes and molecular mechanisms of protein degradation, our analysis incorporated cumulative fluid balance, qualitative muscle assessment, and functional evaluation. The greater magnitude of early muscle deterioration observed in our cohort may reflect the combined influence of several factors commonly present in critically ill patients, including prolonged mechanical ventilation, deep sedation, systemic inflammation, immobilization, illness severity, and fluid accumulation. The relative contribution of each of these factors cannot be determined from the present study.

Notably, cumulative fluid balance showed a strong inverse correlation with muscle strength at awakening (rho = − 0.70, *p* < 0.001) and remained independently associated with lower MRC-SS in multivariable analysis. These findings suggest that muscle alterations in critically ill patients are likely multifactorial. In addition to structural muscle loss, fluid-related changes, prolonged immobilization, deep sedation, illness severity, and other unmeasured factors may all contribute to impaired muscle function.

In a more homogeneous population of patients with severe sepsis and septic shock (median APACHE III = 90), Palakshappa et al. [27] reported a 23% reduction in rectus femoris cross-sectional area during the first week of hospitalization, with an approximately 18% reduction in muscle thickness. Importantly, static measurements of muscle size were not consistently associated with muscle strength at day 7, whereas the rate of muscle loss showed only a moderate correlation with MRC score (rho = 0.51, *p* = 0.03). These findings suggest that reductions in muscle size alone do not fully explain functional impairment in critically ill patients.

The qualitative component becomes particularly relevant when considering the work of Parry et al. [28], who demonstrated significant increases in muscle echo intensity up to 25% alongside reductions in muscle thickness within the first 10 ICU days. They also reported moderate-to-strong associations between ultrasound parameters and functional outcomes at awakening. However, fluid balance was not systematically analyzed in that cohort, leaving unresolved whether part of the increase in echo intensity might reflect changes in extracellular fluid content rather than exclusively irreversible structural degeneration.

In a prospective pilot study in critically ill patients undergoing ultrafiltration, fluid removal over 36 h was associated with significant reductions in muscle echogenicity and subcutaneous tissue thickness, along with correlations between ultrafiltration volume and changes in ultrasound parameters, as well as modest improvements in MRC-SS [29]. These findings suggest that muscle echogenicity may be modulated not only by structural degradation but also by interstitial fluid content. This reversibility supports the hypothesis that part of the ultrasound-detected “muscle quality” deterioration in ICU patients may reflect fluid-related changes in tissue composition rather than purely muscle mass loss.

Importantly, the relationship between extracellular fluid distribution and muscle function has been described in geriatric populations. In community-dwelling elderly women, Hioka et al. reported that an increased total body extracellular-to-intracellular water (ECW/ICW) ratio, measured using bioelectrical impedance analysis, was independently associated with decreased handgrip strength (rho = − 0.453, *p* < 0.001), suggesting that expansion of the extracellular compartment may contribute to impaired muscle function [30]. Similarly, Stanley et al. demonstrated that fluid status can influence ultrasound-derived muscle measurements, particularly those reflecting muscle quality, emphasizing the importance of considering hydration status when interpreting muscle ultrasound findings [31]. In contrast, Hoffmann et al. found no significant association between fluid balance and changes in quadriceps muscle thickness in critically ill children, suggesting that muscle thickness may be less sensitive to fluid shifts than qualitative ultrasound parameters [32]. These observations are consistent with our findings, in which cumulative fluid balance was associated with muscle quality, as assessed by the Heckmatt score, but showed only a weak, non significant association with changes in quadriceps muscle thickness.

The strong negative correlation observed between cumulative fluid balance and MRC-SS (rho = − 0.70, *p* = 0.001), together with the positive association between fluid balance and subcutaneous thickness, suggests that fluid accumulation may influence ultrasound-derived muscle quality parameters. However, these findings should be interpreted cautiously, as fluid balance may also represent a surrogate marker of overall illness severity and other clinical factors that were not fully captured in this retrospective analysis.

The exploratory longitudinal analyses further support these findings. Cumulative fluid balance was associated with changes in the Heckmatt score and subcutaneous tissue thickness from ICU admission to awakening, whereas no significant association was observed with changes in quadriceps muscle thickness. These results suggest that positive fluid balance may predominantly influence qualitative ultrasound characteristics and peripheral tissue edema rather than changes in muscle thickness. This observation reinforces the potential value of muscle quality assessment using the Heckmatt scale, as it may be more sensitive than muscle thickness for detecting fluid-related alterations in critically ill patients.

The additional quantitative grayscale analysis further supports our findings. Grayscale values demonstrated a moderate inverse correlation with muscle strength and a strong positive correlation with cumulative fluid balance, consistent with the qualitative Heckmatt assessment. These findings suggest that both qualitative and quantitative ultrasound measures capture fluid related changes in muscle composition, supporting the robustness of the observed association between positive fluid balance and muscle quality.

This study has several limitations. First, its retrospective observational design precludes any causal inference between cumulative fluid balance and muscle impairment. Although the regression model adjusted for clinically relevant covariates, residual confounding cannot be excluded. Several factors known to influence muscle strength and muscle quality in critically ill patients, including the duration and depth of sedation, use of neuromuscular blocking agents, severity and duration of shock, corticosteroid exposure, nutritional status, and glycemic control, were not systematically evaluated and may have contributed to the observed associations. Consequently, cumulative fluid balance should be interpreted as a potential marker associated with muscle impairment rather than an isolated causal factor. Second, the single center nature of the study may limit the generalizability of the findings, as clinical practices regarding fluid management, sedation strategies, and rehabilitation protocols may vary across institutions.

Third, muscle quality was assessed using the Heckmatt scale, which, although widely used, is a qualitative measure and may be subject to interobserver variability. In this study, all ultrasound assessments were performed by an experienced physiotherapist following standardized acquisition procedures; however, formal assessment of inter-rater reliability was not conducted.

Additionally, quantitative assessment of muscle echogenicity was not available in the present study. Although the Heckmatt scale is a practical and clinically applicable method for evaluating muscle quality, quantitative grayscale analysis provides a continuous and potentially more objective assessment of muscle echogenicity. Future prospective studies incorporating both qualitative and quantitative ultrasound measures may further improve the characterization of muscle alterations in critically ill patients.

Finally, the study population included patients with heterogeneous admission diagnoses, which may have influenced muscle outcomes through different pathophysiological mechanisms. Therefore, external validation in multicenter cohorts with standardized protocols is warranted.

## Conclusion

In critically ill patients, cumulative fluid balance was significantly associated with poorer peripheral muscle quality assessed using the Heckmatt scale and with lower muscle strength. These findings suggest that qualitative ultrasound-derived measures of muscle quality should be interpreted cautiously, as muscle echogenicity may be influenced by fluid-related alterations in tissue composition in addition to intrinsic muscle changes.

## Data Availability

The datasets generated and/or analyzed during the current study are not publicly available due to institutional and privacy restrictions but are available from the corresponding author on reasonable request.
